# Alterations of blood pulsations parameters in carotid basin due to body position change

**DOI:** 10.1038/s41598-018-32036-7

**Published:** 2018-09-12

**Authors:** Alexei A. Kamshilin, Tatiana V. Krasnikova, Maxim A. Volynsky, Serguei V. Miridonov, Oleg V. Mamontov

**Affiliations:** 1 Department of Computer Photonics and Videomatics, ITMO University, 49 Kronverksky Pr., 197101 St. Petersburg, Russia; 2 Department of Circulation Physiology, Almazov National Medical Research Centre, 2 Akkuratova St., 197341 St. Petersburg, Russia; 3 Optics Department, Centro de Investigación Cientfica y de Educación Superior de Ensenada, 3918 Carretera Tijuana-Ensenada, 22860 Ensenada, Baja California Mexico

## Abstract

The velocity of the pulse wave (PWV) propagating through the vascular tree is an essential parameter for diagnostic the state of the cardiovascular system especially when it is measured in the pool of carotid arteries. In this research, we showed for the first time that the time of the blood-pressure-wave propagation from the heart to the face is a function of the body position. Significant asymmetry and asynchronicity of blood pulsations in the facial area were found in a recumbent position. Parameters of blood pulsations were measured by an advanced camera-based photoplethysmography system in 73 apparently healthy subjects. Most likely, observed changes of the blood-pulsation parameters are caused by variations of the arterial blood pressure due to hydrostatic pressure changes, and secondary reaction of blood vessels in response to these variations. Demonstrated feasibility of PWV measurements in the pool of carotid arteries provides considerable advantages over other technologies. Moreover, possibilities of the method to estimate physiological regulation of the peripheral blood flow (particularly, as a response to the gravitational changes) have been demonstrated. The proposed concept allows development of non-invasive medical equipment capable of solving a wide range of scientific and practical problems related to vascular physiology.

## Introduction

Pulse wave velocity (PWV) is a parameter, which measures how fast a pulse of blood pressure propagates through the vascular system. Propagation of the pressure wave from the heart to periphery might be affected by the cardiovascular system, including effects of vascular diseases^1,2^, blood pressure^3,4^, loss of compliance with age^5,6^ and low-frequency autonomic control^7^. Typically, an established methodology to assess PWV is the measurable parameter is estimations of the pulse transit time (PTT), i.e. time difference in the pulse wave arrival in two different sites (usually, in vicinity of carotid and femoral arteries), and measuring the distance between these sites^5,8^. Arterial applanation tonometry, which is achieved by contact pressing of mechanotransducers or piezoelectric sensors to the skin at the carotid, radial, or femoral arteries, is typically used for noninvasive PWV estimations^9^. In alternative technique, the pulse-arrival time in finger (or toe) is measured by photoplethysmography (PPG) to estimate the delay time in respect to the R-wave of simultaneously recorded electrocardiogram (ECG)^2,4,10,11^. It was shown that PWV depends on the blood pressure level: the higher the pressure, the faster the speed of wave travel, and the shorter PTT^2,5,8^. Repeatability of PWV measurements is usually considered as very good^12,13^. In the past few years, several research groups have proposed cuff-less blood pressure (BP) monitoring, which is based on the relationship between systolic BP and PTT, the latter measured by PPG^14–18^. However, only few experimental studies were carried out to study dependence of pulse wave propagation on the body position by single-point, contact-type PPG sensors^19–23^. It remains unclear, what kind of impacts might affect the propagation of the pressure pulse generated by ventricular ejection via the vascular system.

Currently, the most of the studies are devoted to PWV estimations for pulse waves propagating in vessels situated below the heart level (in upper and lower limbs). However, variations of PWV is more informative in the pool of carotid arteries. These are the vessels of this particular pool which are associated with a worsening of the prognosis of patients with essential hypertension^24^. At the same time, in recent years, with the development of the PPG technique, it has become possible to assess PWV in the most important regions. Recent advances of the camera-based (imaging) PPG technique (a modality in which blood pulsations are visualized simultaneously in a large region by means of digital camera^25,26^) allowed accurate measurements of the PTT and its spatial distribution over large areas of the body including the head^27^. The aims of the research were demonstrating a feasibility of the camera-based PPG system for measuring PTT in the pool of carotid arteries and analysis of gravitation influence on this parameter. By using this technique, we measured the spatial distribution of both the blood pulsations amplitude (BPA) and PTT at the subject’s face. It was revealed that both maps are significantly altered after the subject changed the sedentary position for the recumbent one or vice versa.

## Results

### Mapping Pulse Transit Time and Blood Pulsations Amplitude

**Figure 1:**
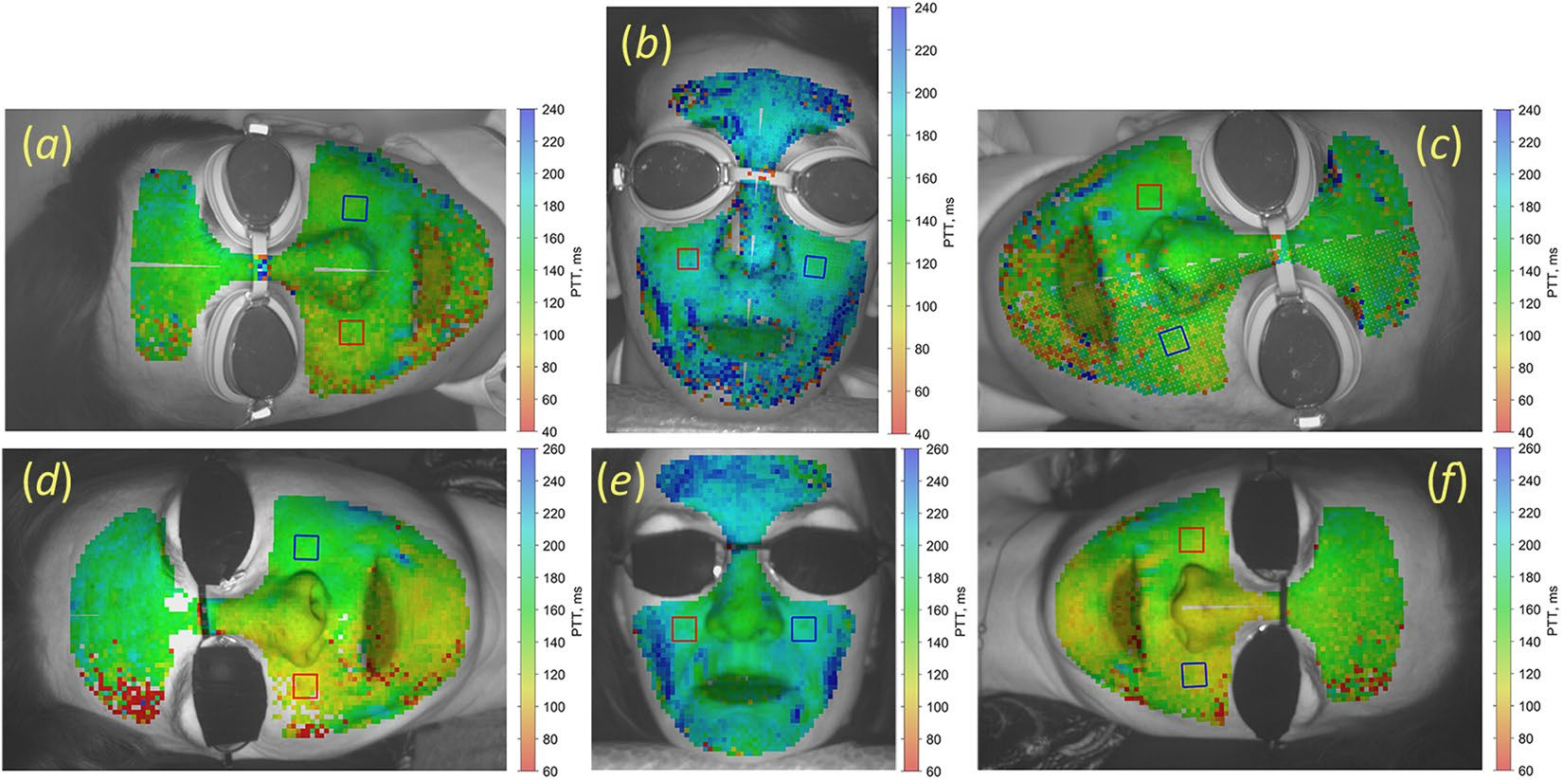
Spatial distribution of PTT overlaid with an image for two subjects in different positions: (**a**) and (**d**), right decubitus of subject A and B, respectively; (**b**) and (**e**), sedentary position; (**c**) and (**f**), left decubitus. The colour scale on the right side of each image shows PTT in milliseconds. Subjects gave their informed consent to the publication of the images in the written form.

**Figure 2:**
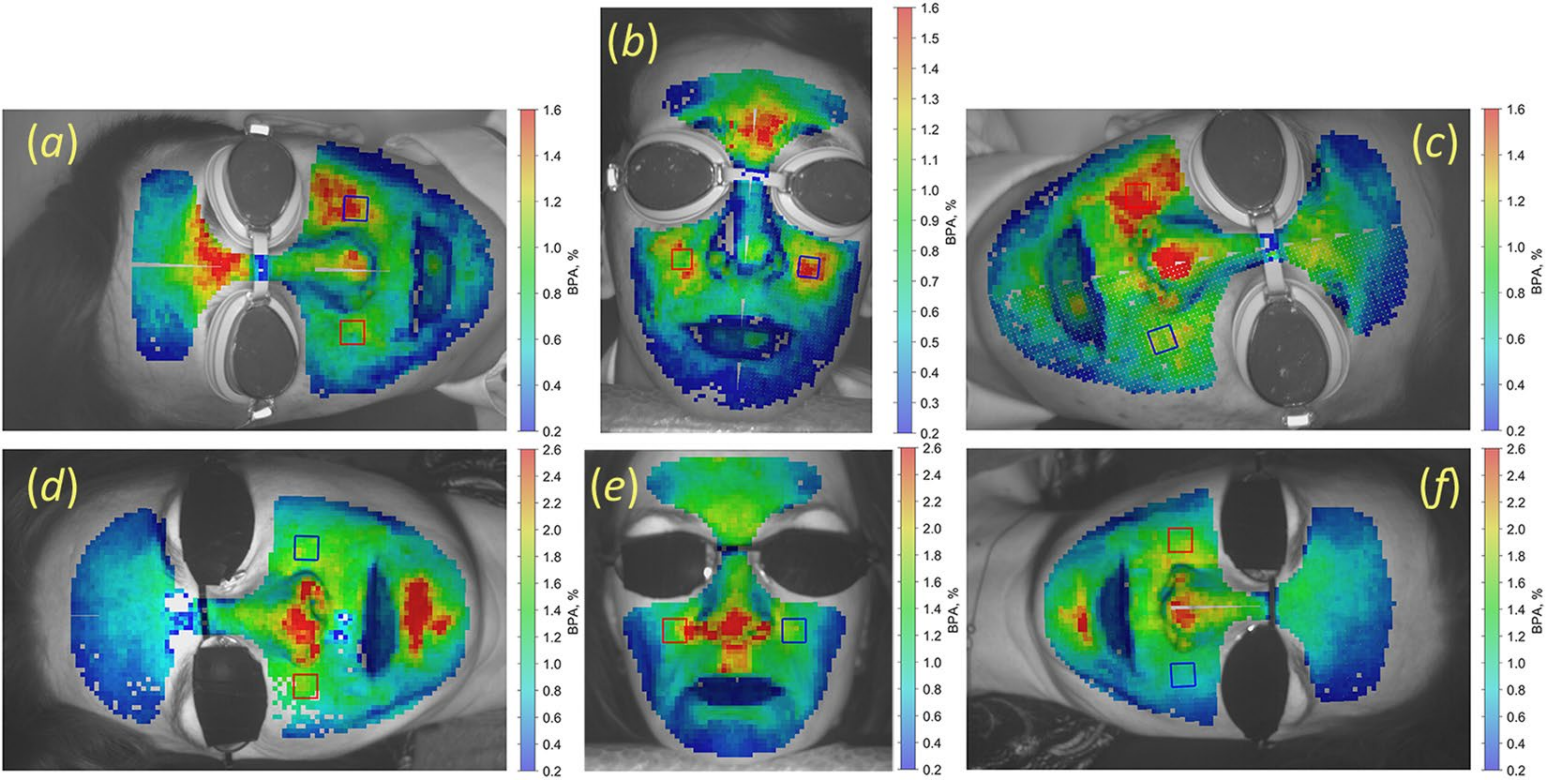
Spatial distribution of BPA for the same subjects as in Fig. 1: (**a**) and (**d**), right decubitus of subject A and B, respectively; (**b**) and (**e**), sedentary position; (**c**) and (**f**), left decubitus. The colour scale on the right side of each image shows BPA in percent. Subjects gave their informed consent to the publication of the images in the written form.

Examples of mapping PTT and blood pulsations amplitude (BPA) calculated for two representative subjects are shown in Figs 1 and 2, respectively. These maps are overlaid on one of the image frames of the subject’s face. The transit time and pulsations amplitude are coded in pseudo-colour with the scale shown in the right side of each map. It is seen that spatial distribution of both the PTT and BPA is heterogeneous in the facial area. Such a heterogeneity was found for all studied subjects. It is worth noting that PTT and BPA patterns are significantly different in sedentary and recumbent positions. It usually takes longer time for the pulse wave to reach the facial area in the sedentary position than it is in any recumbent: the maps are more bluish in the sedentary position (Fig. 1b,d) whereas they are greenish in the recumbent position (Fig. 1a,c,e,f). Note that the PTT scale remains the same for different positions of each subject. The mean value of PTT in the sedentary position measured for the whole cohort was 160 ± 21 ms, whereas it was 124 ± 24 ms in the recumbent position, P < 0.001. Here and below the values are presented as Mean ± Standard Deviation.

Similarly, the amplitude of blood pulsations is also different in sedentary and recumbent positions. The mean BPA for the whole cohort measured in the sedentary position was 1.59 ± 0.75% versus 1.34 ± 0.58% in recumbent positions, P < 0.001. Nevertheless, the spatial pattern of BPA distribution may vary significantly: see Fig. 2a,c. For quantitative estimations of PTT/BPA dependence on the body position, we manually selected bigger ROIs sizing 42 × 42 pixels on the right and left cheeks (shown in Figs 1, 2 by red and blue square, respectively), and averaged the parameters within these ROIs.

Interestingly, no significant difference in blood pulsations parameters were found between younger and older group of subjects, the groups were divided in respect to the median age (24 years) of the whole cohort. Parameter PTT averaged over both cheeks and all positions in the younger group (131 ± 16 ms) was almost the same as in the older (134 ± 18 ms), P = 0.57, and the mean BPA was 1.435 ± 0.39% and 1.431 ± 0.54%, P = 0.97 for younger and older group, respectively. Moreover, no correlation between PTT and the subject’s age (r = −0.03, P = 0.79), and between BPA and age (r = 0.22, P = 0.2) was observed.

### Dependences of PTT and BPA on the body position

**Figure 3:**
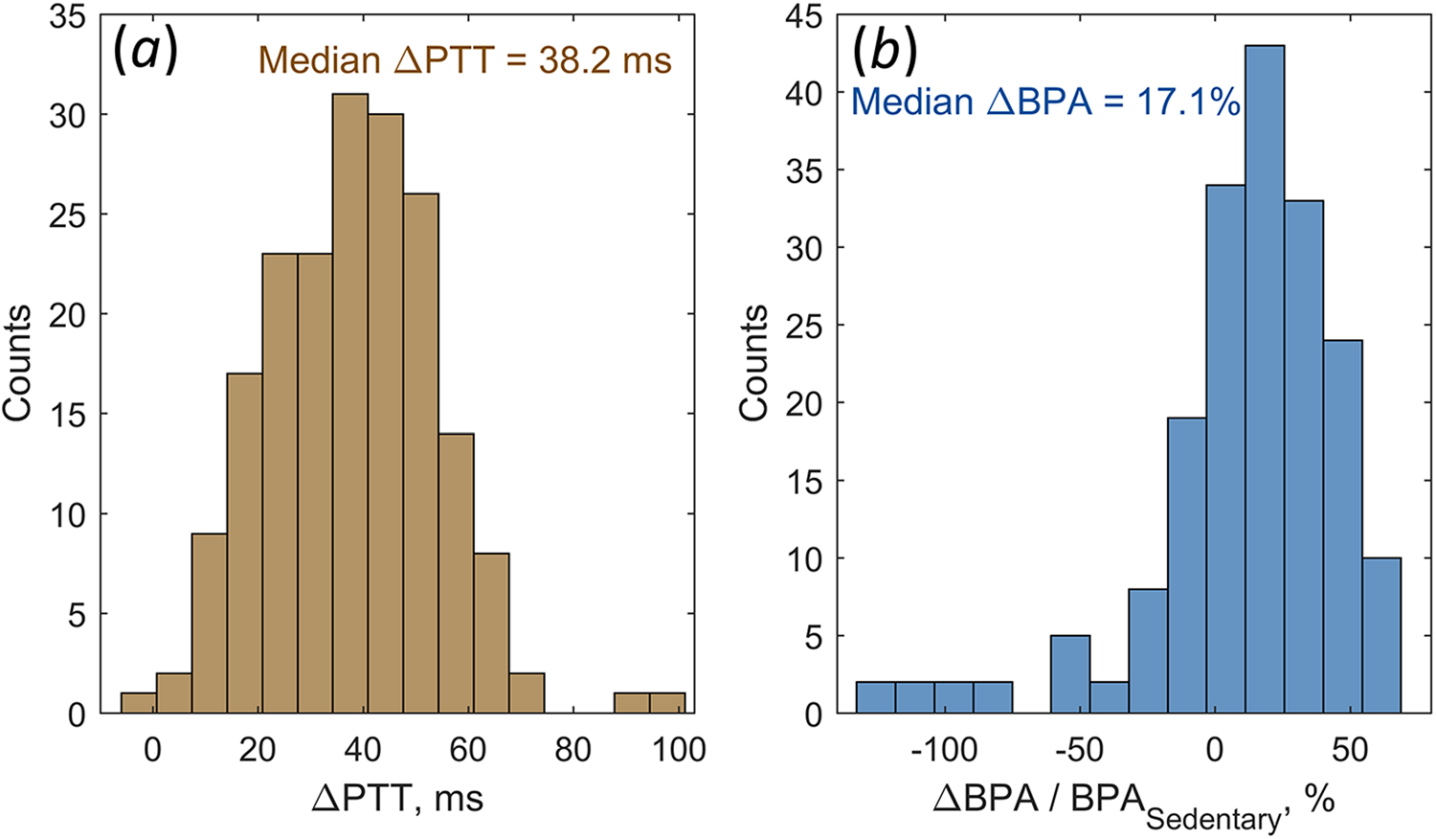
Variations of PTT and BPA caused by the position change. (**a**) Histograms of PTT difference (see Eq. 1) between the sedentary and recumbent positions, and (**b**) relative BPA difference (see Eq. 2) measured for all studied subjects.

Figure 3a shows the histogram of the PTT-difference distribution among the studied participants. This difference between PTT measured in the sedentary and recumbent positions was calculated separately for left (${\rm{\Delta }}PT{T}_{L}$) and right ($\Delta PT{T}_{R}$) decubitus as

1 $$\begin{array}{c}{\rm{\Delta }}PT{T}_{L}=0.5(PT{T}_{S}^{R}+PT{T}_{S}^{L}-PT{T}_{L}^{R}-PT{T}_{L}^{L}),\\ {\rm{\Delta }}PT{T}_{R}=0.5(PT{T}_{S}^{R}+PT{T}_{S}^{L}-PT{T}_{R}^{R}-PT{T}_{R}^{L}),\end{array}$$

where $PT{T}_{S}^{R}$ and $PT{T}_{S}^{L}$ are the transit time measured in the sedentary position within the big ROIs at the right and left cheeks, respectively; $PT{T}_{L}^{R}$, $PT{T}_{L}^{L}$ and $PT{T}_{R}^{R}$, $PT{T}_{R}^{L}$ are the similar parameters measured in the left and right decubitus, respectively. The histogram in Fig. 3a includes both Δ*PTT*_*L*_ and Δ*PTT*_*R*_. It is clearly seen that only one subject in one recumbent position has the PTT slightly longer than in the sedentary position. For the most of participants the situation was inversed: the pulse wave propagated faster in the recumbent position. The median decrease of PTT in the recumbent position was 38.2 ms, and Δ*PTT* larger than 14 ms was observed in 94% of subjects. Note that Δ*PTT* of 38 ms corresponds to about 25% decrease of the PTT measured in the sedentary position.

The difference of the pulsation amplitude Δ*BPA* was also estimated for the left (Δ*BPA*_*L*_) and right (Δ*BPA*_*R*_) decubitus positions separately. In contrast to estimations of Δ*PTT* it was calculated in the relative units:

2 $$\begin{array}{c}{\rm{\Delta }}BP{A}_{L}=\frac{BP{A}_{S}^{R}+BP{A}_{S}^{L}-BP{A}_{L}^{R}-BP{A}_{L}^{L}}{BP{A}_{S}^{R}+BP{A}_{S}^{L}}100 \% ,\\ {\rm{\Delta }}BP{A}_{R}=\frac{BP{A}_{S}^{R}+BP{A}_{S}^{L}-BP{A}_{R}^{R}-BP{A}_{R}^{L}}{BP{A}_{S}^{R}+BP{A}_{S}^{L}}100 \% ,\end{array}$$

Here $BP{A}_{S}^{R}$ and $BP{A}_{S}^{L}$ are the blood pulsation amplitude measured in the sedentary position within the same big ROIs at the right and left cheeks, respectively; $BP{A}_{L}^{R}$, $BP{A}_{L}^{L}$ and $BP{A}_{R}^{R}$, $BP{A}_{R}^{L}$ are the similar parameters measured in the left and right decubitus position, respectively. The histogram of Δ*BPA* distribution among the studied participants is shown in Fig. 3b. One can see that the mean amplitude of blood pulsations in the cheeks is decreasing for the majority of studied participants (76.6%) when a subject changes the sedentary position for a recumbent. The median relative change of BPA observed in the studied cohort was 17.1%. Nevertheless, there are some subjects showing increase of mean BPA in a recumbent position as compared to the sedentary.

### Asymmetry and asynchronicity of blood pulsations

**Figure 4:**
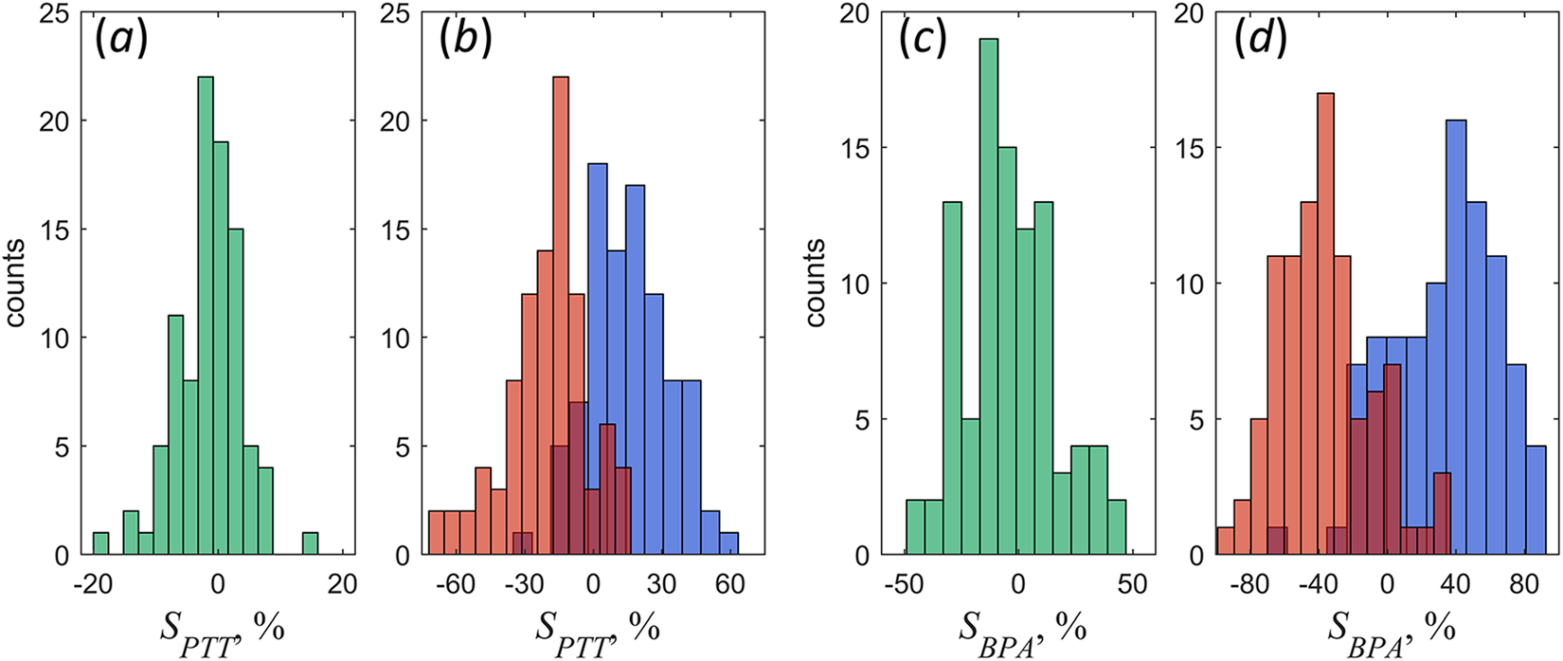
Degree of asymmetry and asynchronicity of PTT and BPA in different positions. Histograms of *S*_*PTT*_ and *S*_*BPA*_ distributions for subjects in the sedentary position are shown in panels (**a**) and (**c**), respectively. Histograms of *S*_*PTT*_ and *S*_*BPA*_ in the recumbent position are in panels (**b**) and (**d**), respectively. Red bins in the panels (**b**) and (**d**) were obtained for subjects in the right decubitus, whereas blue bins were measured in the left decubitus.

Additionally to changes of the mean BPA/PTT with the body position, our experiments revealed significant variations in spatial distribution of these parameters. PTT and BPA maps of the facial area have individual spatial distribution of these parameters for each subject as one can see in Figs 1 and 2. Sometimes these distributions are clearly asymmetric (for example, Figs 1d and 2a, or Fig. 2c). Moreover, the degree asymmetry varies with the change of position. In the BPA map of Fig. 2a, the amplitude of pulsations in the left cheek (1.40%) is much higher than in the right cheek (0.86%). In contrast, for the same person in other recumbent position (Fig. 2c) BPA in the left cheek (1.09%) becomes smaller than in the right (1.63%). Asymmetry of PTT maps means asynchronicity of blood pulsations in the right and left sides. For quantitative estimation of the degree of asymmetry and asynchronicity, we calculated the respective coefficients *S*_*PTT*_ and *S*_*BPA*_ as

3 $$\begin{array}{c}{{S}}_{{PTT}}=\frac{2({PT}{{T}}^{{R}}-{PT}{{T}}^{{L}})}{{PT}{{T}}^{{R}}+{PT}{{T}}^{{L}}},\\ {{S}}_{{BPA}}=\frac{2({BP}{{A}}^{{R}}-{BP}{{A}}^{{L}})}{{BP}{{A}}^{{R}}+{BP}{{A}}^{{L}}},\end{array}$$

where *PTT*^*R*^ and *BPA*^*R*^ are measured in the big ROIs at the right cheek, whereas *PTT*^*L*^ and *BPA*^*L*^ are for the left cheek. The parameters *S*_*PTT*_ and *S*_*BPA*_ were measured for each subject in three positions: sedentary, right and left decubitus. Histograms of the results obtained for the whole cohort of the participants are shown in Fig. 4.

As one can see, in the sedentary position both histograms (Fig. 4a for degree of asynchronicity and Fig. 4c for degree of asymmetry) are centred on zero. It means that blood pulsations are symmetrical and synchronous in the right and left cheeks for the most of healthy subjects with equally probable deviations to the right or left side depending on the physiological features of the subject. In contrast, we observed significant increase of both the degree of asynchronicity and asymmetry when the subject is in the recumbent position. It is clearly seen in Fig. 4b,d that the histograms for left and right decubitus (shown by red and blue bins, respectively) are much wider than for the sedentary, and their distributions are not centred at zero values. Median degree of PTT asynchronicity in the right decubitus is −0.18, whereas in the left decubitus it is 0.15. Recalling that the left cheek is up in the right decubitus while the right cheek is up in the left decubitus and using *S*_*PTT*_ definition in Eq. 3, we conclude that PTT measured in the upper cheek is usually longer than that in the lower one for any recumbent position. PTT asynchronicity can be clearly seen in Fig. 2d,f as well. For all subjects in recumbent positions, the mean PTT measured in the upper cheek was 133 ± 22 ms in contrast with 112 ± 21 ms in the lower cheek, P < 0.001.

Spreading of *S*_*BPA*_ histogram (Fig. 4d) is even larger than of *S*_*PTT*_ showing the median degree of asymmetry in the right decubitus of −0.39, whereas it is 0.35 in the left decubitus. Therefore, the amplitude of blood pulsations with higher probability is larger in the upper cheek in any recumbent position as it is clearly seen in BPA maps in Fig. 2a,c. In recumbent positions, the mean BPA in the upper cheek was 1.51 ± 0.57% versus 1.07 ± 0.43% in the lower cheek, P < 0.001.

## Discussion

According to the recent model proposed by our group^28,29^, interaction of the polarized green light with skin integument allows assessment of the erythrocytes speed in capillaries of the papillary layer. Recordings of the pulsatile components originating from the pulse wave arrived to the facial area allows us mapping the spatial distribution of both the amplitude and dynamic parameters of the capillary blood flow^28^. Due to described technological advances, these maps were calculated with high spatial and temporal resolution. Noteworthy that synchronization of the peripheral pulse wave with ECG allowed us to measure PTT with high accuracy in regions, which were previously inaccessible for such measurements. At the same time, blood flow changes in the pool of carotid arteries are the most informative in the prognosis estimation for patients with cardiovascular disease. Moreover, our approach is capable to carry out dynamic observations of the vascular parameters variations, which makes it an indispensable tool in the study of the physiological mechanisms of blood circulation regulation.

We assume that the PPG waveform under green illumination originates from the upper capillary level^28,29^. Considering the small length of capillaries (about 1 mm), the time delay of the pulse wave between the arteriole and capillary is negligible compare to the time needed to arrive the arteriole. Therefore, modulation of the erythrocytes speed in capillaries accurately enough describes the shape of the pulse wave in the point of measurements. In recent comparative measurements of the erythrocytes speed and PPG waveforms, the high correlation between these waveforms was reported^28^ suggesting smallness of the delay between them. However, one may assume existence of additional time delay between erythrocytes speed and light intensity modulation due to still debatable mechanism of light intensity modulation in capillaries. Therefore, more detailed study is needed to deeper understand the mechanism of light modulation.

Our experiments have shown that the camera-based PPG technique is capable to measure PTT between the heart and face. Therefore, we demonstrated feasibility to assess the velocity of the pulse wave propagating in the pool of carotid arteries. This provides considerable advantages over other technologies in estimating the PWV parameter. Moreover, possibilities of the method to estimate physiological regulation of the peripheral blood flow (particularly, as a response on the gravitational changes) have been demonstrated.

Observed decrease of PTT in recumbent position (Fig. 3a) corresponds to increase of the pulse wave velocity assuming the distance of the pulse wave propagation from the heart to the face gets negligible changes after the position change. Corresponding increase of PWV can be explained by increase of the systolic BP in the recumbent position due to the gravitational effects. Position of the head in respect to the heart in the sedentary position is in average by 20 cm higher than in the recumbent position. This difference of hydrostatic pressure corresponds to 15 mmHg. Therefore, we may assume that the systolic BP in the sedentary position is smaller than in the recumbent. This assumption is supported by our oscillometric measurements of BP carried out with 10 selected participants in lateral decubitus and supine positions. In the decubitus, the cuff on the shoulder was above the heart level by 15 cm in average, whereas in the supine position, the cuff was at the heart level. The mean systolic BP was measured as 109 ± 17 mmHg in the decubitus and 121 ± 12 mmHg in the supine showing the smaller BP in the higher position of the expected difference of 12 mmHg. However, the relative change of BP (10–13%) was smaller than the measured change of PTT. According to the histogram of Fig. 3a, PTT is decreasing by more than 41 ms (which corresponds to more than 25% of the relative change) in 44% of studied subjects. Moreover, dispersion of the hydrostatic pressure difference (related to the subject’s height of 173.9 ± 7.4 cm, the relative dispersion of 4.3%) was much smaller than the observed dispersion of PTT difference 37.9 ± 16.2 ms, the relative dispersion of 42.7%.

Hydrostatic pressure difference can be estimated more accurately between the big ROIs in right and left cheeks in decubitus position, which is about of 7.5 cm in average. It corresponds to the pressure difference of 5.7 mmHg, which is less than 5% of the relative change of the systolic blood pressure. Lower BP in the upper cheek leads to longer PTT, which is in accordance with the observed asynchronicity of blood pulsations, see Figs 1 and 4b. However, mean degree of asynchronicity in the decubitus position is 0.181 corresponding to 18.1% of the relative change of PTT. Moreover, 22% of subjects show the relative change of PTT in decubitus exceeded 31%, which is much higher than the expected hydrostatic pressure difference.

As commonly accepted in photoplethysmography^30,31^, the parameter BPA is the fraction of pulsatile variations over the mean signal intensity (see the next Section “Methods”). Therefore, change of this parameter in different body position might be attributed with either pulsatile or mean signal variations. In the current experiments, it was technically difficult to keep the same intensity of illumination on the cheeks when subject change his position. Nevertheless, recent study of BPA evolution during capsicum plaster application (with the stable skin illumination by the green light) has revealed that the relative increase of the pulsatile component due to precapillary sphincters opening caused by capsaicin is 25 times higher than concomitant decrease of the mean signal^32^. Therefore, we suppose that in our case change of the pulsatile component is the main reason of BPA alteration as well.

In view of these findings, possible explanation of the position-dependent dynamics of the PTT and BPA parameters by change in the tone of the sympathetic nervous system does not seem convincing. First, sympathetic vascular tone varies symmetrically on both sides of the body. Second, the tone is diminishing in the decubitus position resulting in deceleration of the pulse wave^33^, whereas we have observed significant acceleration of PWV: 3.38 ± 0.83 m/s in recumbent positions versus 2.54 ± 0.36 m/s in the sedentary, P < 0.001. Therefore, we hypothesize that observed changes of PTT and BPA are caused by two different factors. These factors are (i) variations of arterial blood pressure due to hydrostatic pressure changes, and (ii) secondary reaction of blood vessels in response to these variations. Both factors can affect nonlinearly the measured parameters.

Our experiments demonstrated that the camera-based PPG system is capable to carry out dynamic observation how the vascular parameters are changing under the influence of various physiological impacts. Particularly, a gravitational dependence of the pulse waves reaching the head has been revealed. On the one hand, observed variations of PTT with changes of a body’s position should be taken into account while calibrating future noncontact systems of BP monitoring using PTT. On the other hand, camera-based PPG system could be used as a simple and robust device for assessment of the vasomotor regulation mechanisms. This non-invasive and simple in implementation technique can be useful for (i) significant facilitation of the population study of vascular stiffness, (ii) clarification the mechanisms of vascular tone regulation in the carotid basin, and (iii) assessment the regress of the indicators deviation from the norm caused by therapeutic and surgical interventions. Moreover, the proposed concept will have important applications since it allows development of non-invasive medical equipment capable of solving a wide range of scientific and practical problems related to vascular physiology.

## Methods and Subjects

### Participants

The study involved 73 apparently healthy subjects (33 females and 40 males) between 18 and 65 years (29.2 ± 12.0) years. Persons with any neurologic, cardiovascular or skin diseases were not invited to participate in this study. This study was conducted in accordance with ethical standards presented in the 2013 Declaration of Helsinki. The study plan was approved by the research ethical committee of the Almazov National Medical Research Centre prior the experiments. All subjects provided their informed consent in the written form for participation in the experiment and for the publication of identifying information/images in an online open-access publication.

### Measurement system

**Figure 5:**
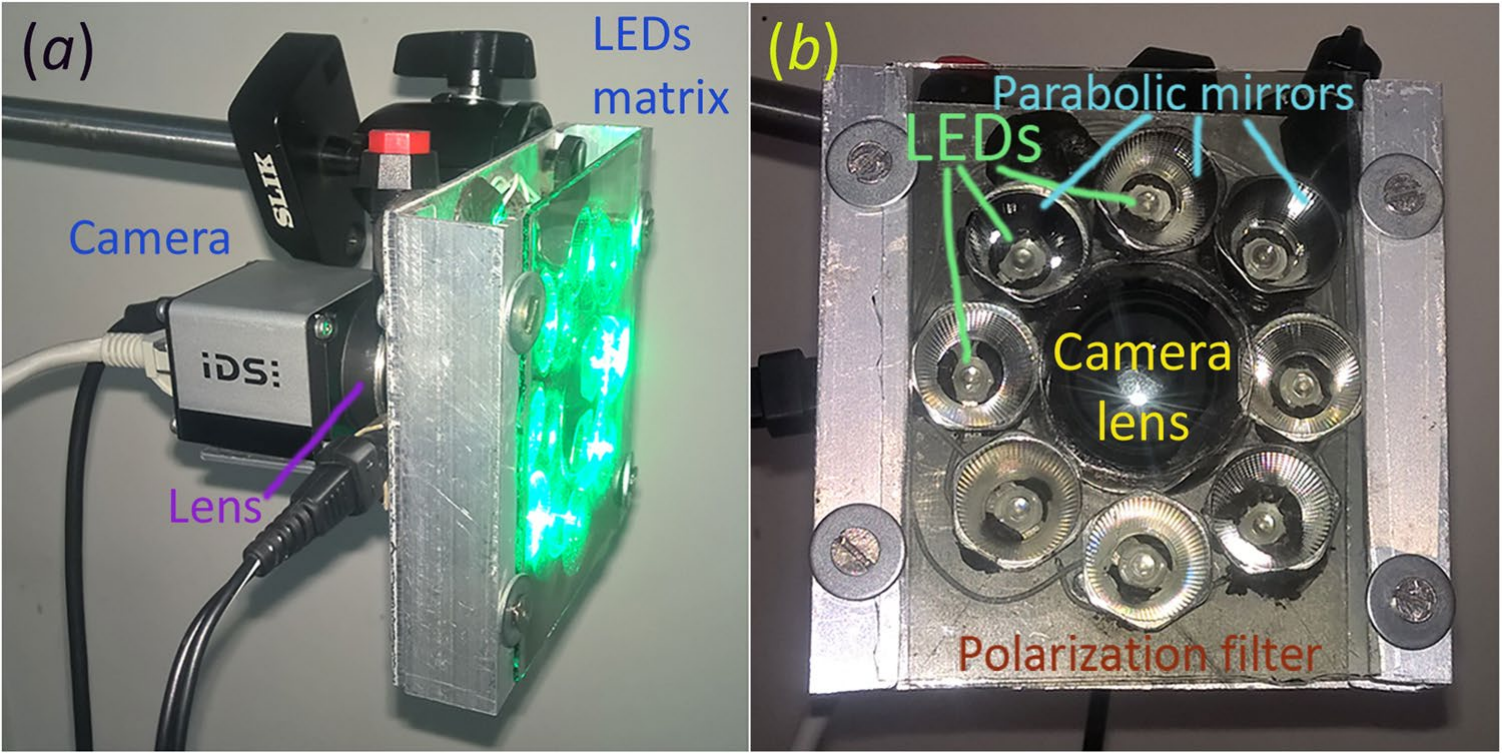
Photograph of the custom-made IPPG system. (**a**) General view of the system with LEDs on. (**b**) Top view of the system showing camera lens, the LEDs matrix with parabolic mirrors, and polarizing filter.

A custom-made camera-based PPG system was used to collect the data from facial area of a subject. The system consisted of a digital monochrome CMOS camera (8-bit model GigE uEye UI-5220SE of the Imaging Development Systems GmbH) and an illuminator with a polarization filter. A photograph of the system is shown in Fig. 5. For illuminating subject’s face we designed and built a matrix which holds 8 light emitting diodes (LED) operating at the wavelength of 530 nm with the spectral bandwidth of 40 nm (green light) and the power of one watt per LED. Each LED was placed inside a separate parabolic mirror, which diminished initial divergence of light flux emerged from the LED (see Fig. 5b). All LEDs were assembled around the camera lens. The polarization filter consists of inner circle and outer part with mutually orthogonal transmission axes. Such polarization filtration reduces the skin specular reflections and motion artefacts influence on the detected PPG waveform^34^. Light emitted from LEDs passes through the outer polarizing part whereas the inner circle serves as a filter for light received by the camera lens. Position of LEDs with parabolic mirrors were adjusted to provide uniform illumination of an area centred with the axis of observation at the distance between 0.2 and 1.5 meter from the camera lens. The whole system was aligned so that perpendiculars to surfaces of both cheeks and forehead were as close to the optical axis of the camera lens as possible. In this geometry the light was directed almost orthogonally to cheeks and forehead thus diminishing the negative influence of the ballistocardiographic effects^31^.

All videos were recorded at 39 frames per second with pixel resolution of 752 × 480 and saved frame-by-frame in PNG format in the hard disk of a personal computer. The distance between the camera lens and subject’s face under study was about 0.7 m. Experiments were carried out in a black-out laboratory room providing that the intensity of the LED illumination at the subject’s face was at least ten times higher than the intensity of the ambient light. ECG was recorded simultaneously with video frames by a digital electrocardiograph. Synchronization accuracy between the electrocardiograph and the camera was better than 1 ms. To implement ECG recordings, disposable electrodes were attached to the left and right wrists with the reference electrodes on the legs.

### Study design

The video recordings of each subject’s face was carried out in three different positions: one sedentary and two recumbent (right and left decubitus). In sedentary position, subject was asked to sit comfortably and lean his head on a properly adjusted support. In recumbent positions, his head was on a pillow. The video recordings was implemented during 30–40 s in each position. After changing the position, a subject relaxed during 5 minutes before the recording in other position was started. Before video and ECG data recordings in each position, we measured the blood pressure (BP) of the participant by conventional oscillometric cuff-based BP device (A&D Medical).

### Data processing

**Figure 6:**
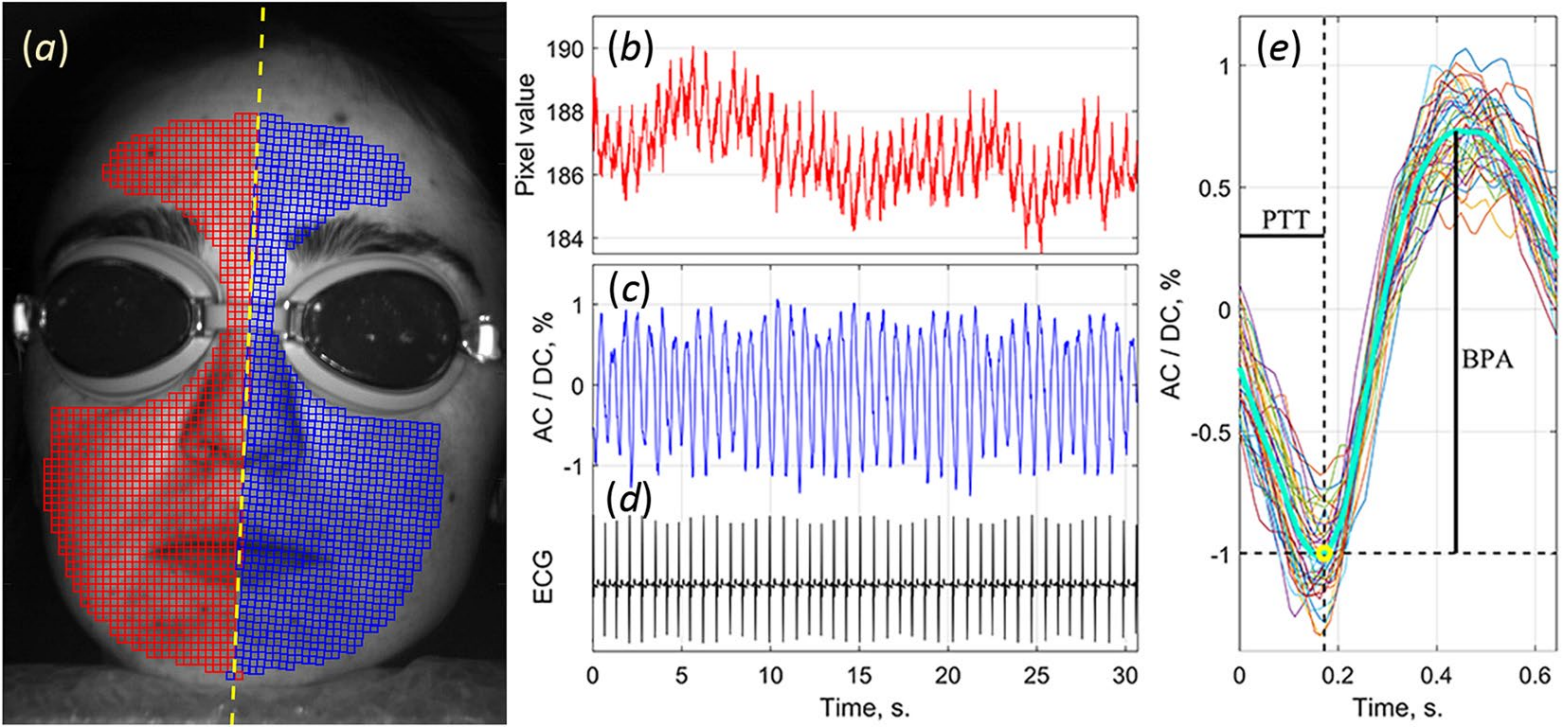
Selecting the ROIs positions on the recorded face image (**a**) and typical example of the PPG waveform from one of the ROIs in the right cheek. (**b**) Raw signal averaged in the ROI of 7 × 7 pixels without any filtration. (**c**) PPG waveform as AC-to-DC ratio after motion-compensating procedure and low-pass filtering. (**d**) ECG signal synchronously recorded with video frames. (**e**) One-cardiac-cycle waveform (thick green line) obtained after averaging of particular waveforms (thin colour lines) over 30 cycles. Subject gave his informed consent to the publication of the image in the written form.

All recorded video frames from the cameras were processed off-line by using custom software implemented in the MATLAB® platform. First, we manually designated a symmetry line in the recorded image of the subject’s face and selected an area for analysis on the right side of the face. This area was completely covered by small regions of interest (ROI) sizing 7 × 7 pixels, which approximately corresponds to 2 × 2 mm^2^ in the facial area. Each ROI was chosen to have a common border with adjacent ROIs without overlapping. Positions of the ROIs in the left side of the face image were chosen to be symmetrical with the ROIs in the right side in respect to the symmetry line as shown in Fig. 6a. Second, we calculated PPG waveform as frame-by-frame evolution of average pixel value in every chosen ROI. An example of raw waveform (without any filtering) is shown in Fig. 6a. Typically, it consists of alternating component (AC), which follows to the heartbeats, and slowly DC varying component. Both components are proportional to the incident light intensity^26^. To compensate unevenness of illumination, we calculated AC/DC ratio, deduced the unity from the calculated ratio, and inverted the sign. These transformation are typical in photoplethysmography providing the waveform to correlate positively with variations of arterial blood pressure^29,30^.

Abovementioned steps are similar to the algorithm described in our recent paper^27^ dealing with PTT calculations from the camera-based PPG signals. Aiming further increase of the algorithm reliability, we added here a pre-processing step for compensation of involuntary face motions during video recordings. Considering that different parts of the face are displaced stochastically and heterogeneously, we divided the whole image on the segments of 64 × 30 pixels, and compensated the motion of each segment independently. We assumed that the signal variations have two components: PPG and the motion-related parts. The motion-related component is proportional to the image gradient and lateral offset. The lateral offset was estimated in every segment by optical flow algorithm using gradient method^35,36^ and then the motion-related signal component was reconstructed and subtracted from the original signal.

All PPG waveforms were filtered to remove noise and DC components by means of a band-pass filter (0.12–20 Hz), which was used with the ***filtfilt*** function in Matlab to perform zero-phase digital filtering of the waveforms. An example of the filtered signal is shown in Fig. 6c along with the simultaneously recorded ECG signal (Fig. 6d). As seen, each oscillation of the waveform follows the R-peak of ECG signal. These oscillations are plotted together in Fig. 6e by thin coloured lines so that each R-peak is at the beginning of the time scale. Thick greenish line in Fig. 6e shows a mean waveform obtained by averaging the filtered one-cardiac-cycle oscillations during the 30 s. The transit time of the pulse wave, PTT, was calculated as the time delay between the R-peak (zero of the abscissa axis in Fig. 6e) and the minimum of the mean waveform (yellow circle in Fig. 6e) because the latter corresponds to the beginning of the anacrotic wave with fast blood-pressure increase. Amplitude of blood pulsations, BPA, is estimated as the difference between the maximum and minimum values of the mean PPG waveform (see Fig. 6e). Parameters PTT and BPA were calculated for each selected ROI thus allowing us their mapping in the image of the whole face.

Depending on the heart rate, from 30 to 50 cardiac cycles were used to estimate the PTT and BPA parameters in each ROI sizing 7 × 7 pixels. The standard deviation of the mean PTT in each ROI varied from 20 to 40 ms. Consequently, the standard error of the PTT estimation was the square root of the number of cycles smaller and varied from 3 to 8 ms. Similarly, the relative error of the BPA estimations was between 3.6 and 9.7%.
